# Optimization, Structural Characterization, and Bioactivities of Polysaccharides from *Rosa roxburghii* Tratt Fruit Using Enzyme-Assisted Extraction

**DOI:** 10.3390/foods14142423

**Published:** 2025-07-09

**Authors:** Qing Chen, Yue Zhang, Siyuan Zheng, Siming Zhu, Chao Li

**Affiliations:** School of Food Science and Engineering, South China University of Technology, Guangzhou 510641, China; chenqing28532@163.com (Q.C.); zy17733135750@163.com (Y.Z.); zhengsiyuan0306@163.com (S.Z.)

**Keywords:** polysaccharides, antioxidant, immunomodulatory

## Abstract

This study aimed to optimize the enzyme-assisted extraction of polysaccharides (RTFPs) from *Rosa roxburghii* fruit using response surface methodology. Under the optimal extraction conditions, the yield of RTFPs reached 14.02%, which was close to the predicted value of 13.96%. The primary structural characteristics and the antioxidative and immunomodulatory activities of RTFPs were also examined. Structural characterization revealed that RTFPs comprise 36.38% neutral sugar, 48.83% uronic acid, and 7.29% protein. Their heteropolysaccharide structure features two distinct molecular weight fractions (1.87 × 10^5^ Da and 4.75 × 10^3^ Da) and a monosaccharide composition dominated by glucose (38.93%), arabinose (20.66%), galactose (20.58%), galacturonic acid (10.94%), and xylose (6.52%). Antioxidant assays demonstrated potent radical scavenging activity, with IC_50_ values of 11 μg/mL (DPPH) and 150 μg/mL (ABTS), comparable to conventional antioxidants. Immunomodulatory studies on RAW264.7 macrophages revealed that RTFPs (100–400 μg/mL) significantly enhanced phagocytosis by 12.61–76.63% and stimulated the secretion of nitric oxide (NO) and tumor necrosis factor-α (TNF-α). These bioactivities are attributed to RTFPs’ high uronic acid content, moderate molecular weight distribution, unique monosaccharide profile, and highly branched conformation.

## 1. Introduction

*Rosa roxburghii* Tratt (*R. roxburghii*), a perennial shrub endemic to southwestern China, grows at elevations ranging from 500 to 2500 m. Its fruit has been historically valued in Chinese traditional practices as both a functional food and therapeutic agent due to its abundant bioactive constituents, including triterpenoids, flavonoids, and polysaccharides. Recent studies have increasingly highlighted the therapeutic potential of *R. roxburghii* fruit polysaccharides, demonstrating significant hypoglycemic, hypolipidemic, anticancer, anti-inflammatory, and gut microbiota-modulating properties [1,2,3]. In recent years, the growing interest in the development and application of *R. roxburghii* fruit has led to the introduction of various food and nutraceutical products in China, such as health supplements, beverages, and functional foods that leverage the plant’s beneficial properties. However, there is limited research on the extraction technology of *R. roxburghii* fruit polysaccharides, which can significantly influence their yield, structure, and biological activities. Therefore, it is essential to explore and study effective extraction methods that maximize polysaccharide yield while preserving their bioactive components.

While conventional hot water extraction remains prevalent in industrial polysaccharide isolation due to its simplicity and cost-effectiveness, it has inherent limitations, including suboptimal yields (typically <5%), prolonged processing times (4–6 h), and thermal degradation risks [4]. Enzyme-assisted extraction has emerged as a sustainable alternative, utilizing cell wall-degrading enzymes (e.g., cellulase, pectinase) to enhance polysaccharide release by targeting cell wall disruption [5]. Comparative studies have shown substantial advantages over traditional methods: Wang et al. achieved a 163% increase in yield (10.53% vs. 4.21%) for *Acanthopanax senticosus* polysaccharides with reduced processing time [6], while enzyme-extracted polysaccharides from *Agaricus blazei Murrill* and lotus leaf demonstrated superior antioxidant activities compared to hot water extracts [7,8]. These findings suggest that enzyme-assisted methods not only improve extraction efficiency but may also preserve or enhance bioactive conformations through mild processing conditions. Given these advancements, optimizing enzyme-assisted extraction techniques for *R. roxburghii* fruit polysaccharides holds great potential for maximizing their health benefits.

In this study, we aimed to optimize the enzyme-assisted extraction of polysaccharides (RTFPs) from *R. roxburghii* fruit. The extraction conditions were optimized through single-factor experiments, followed by response surface methodology (RSM). The primary structural characteristics and antioxidative and immunomodulatory activities of the RTFPs were determined through in vitro antioxidant assays and immunomodulation tests. This research will provide valuable insights into the efficient extraction and potential applications of *R. roxburghii* fruit polysaccharides in functional foods.

## 2. Materials and Methods

### 2.1. Materials and Chemicals

*Rosa roxburghii* fruits were obtained from Guizhou Xinyang Agricultural Science and Technology Development Co., Ltd. (Bijie, China). The fruits were air-dried at 45 °C for 48 h, followed by desiccation, crushing using a laboratory mill (FW 135, Taisite, Tianjin, China), and sieving through a 40-mesh sieve to obtain the powdered sample. Cellulase (50 U/mg) was purchased from Shanghai Yuanye Biotechnology Co., Ltd. (Shanghai, China). 2,2-Diphenyl-1-picrylhydrazyl (DPPH) and 2,2′-azino-bis(3-ethylbenzothiazoline-6-sulphonic acid) (ABTS) were obtained from Sigma-Aldrich Chemical Co. (St Louis, MO, USA). Dulbecco’s modified Eagle’s medium (DMEM), fetal bovine serum (FBS), streptomycin, and penicillin were purchased from Gibco Life Technologies (Grand Island, NY, USA). The monosaccharide standards, including arabinose, galactose, glucose, xylose, mannose, fucose, galacturonic acid, and glucuronic acid, were purchased from Shanghai Aladdin Biochemical Technology Co., Ltd. (Shanghai, China). All reagents and chemicals used were of analytical grade or higher.

### 2.2. Single-Factor Experimental Design

A single-factor experimental design was employed to investigate the effects of extraction time, liquid-to-solid ratio, extraction temperature, and extraction pH on the yield of RTFPs, aiming to determine the optimal extraction conditions. The extraction conditions were as follows: cellulase concentration was maintained at 2% (*w*/*w*), with extraction time set at 50, 70, 90, 110, and 130 min; solid-to-liquid ratios were set at 10, 20, 30, 40, and 50 mL/g; extraction temperatures were set at 30, 40, 50, 60, and 70 °C; and extraction pH was adjusted to 3, 4, 5, 6, and 7. Each experiment was conducted in triplicate.

### 2.3. Response Surface Optimization

Based on the results from the single-factor experiments, three factors were identified as having the most significant effects on the yield of RTFPs: liquid–solid ratio (A), extraction pH (B), and extraction temperature (C). These factors were selected for optimization using a three-factor, three-level Box–Behnken central composite design. Design-Expert V13 response surface software was employed for the analysis, and the levels and codes of the factors are detailed in Table 1.

### 2.4. Enzyme-Assisted Extraction of Polysaccharides from R. roxburghii Fruit (RTFPs)

The extraction of RTFPs was performed using a previously reported method with minor modifications [9]. Briefly, the powder of *R. roxburghii* fruit was mixed with 95% ethanol at a solid-to-liquid ratio of 1:6 (g/mL) and stirred for 2 h. This process was repeated five times. After centrifugation (4000 rpm, 10 min), the residue was dried at 45 °C for 24 h to allow complete evaporation of the ethanol. The pretreated powder was then extracted with deionized water under conditions defined by the single-factor experimental parameters, and cellulase (2%, *w*/*w*) was added. The extraction solution was subsequently heated in a water bath at 90 °C for 5 min to deactivate the enzyme. After centrifugation (4000 rpm, 5 min), the supernatant was collected and concentrated to approximately one-fourth of its original volume. The concentrated solution was deproteinized using Sevag reagent (chloroform/*n*-butanol 4:1, *v*/*v*). This procedure was repeated until no white protein residue remained at the interface of the chloroform and *n*-butanol solution. The residue of Sevag reagent was removed from the polysaccharide solution using a rotatory evaporator under reduced pressure at 45 °C. Anhydrous ethanol was then added until the ethanol concentration in the mixture reached 80% (*v*/*v*), and the solution was allowed to precipitate at 4 °C for 12 h. After centrifugation (5000 rpm, 10 min), the precipitate was washed with anhydrous ethanol and acetone, then dissolved in deionized water and lyophilized to obtain the polysaccharide, named RTFPs. The yield of RTFPs was calculated using the following Equation (1):

(1) $$\mathrm{Yield}\;(\%)=\frac{\mathrm{dry}\;\mathrm{weight}\;\mathrm{of}\;\mathrm{RTFP}s}{\mathrm{dried}\;\mathrm{weight}\;\mathrm{of}\;R.\;\mathrm{roxburghii}\;}\times \;100\%$$

### 2.5. Hot Water Extraction of Polysaccharides from R. roxburghii Fruit (W-RTFPs)

*R. roxburghii* powder was mixed with deionized water at a liquid-to-solid ratio of 38.7 mL/g and subjected to water bath treatment at 90 °C for 90 min. After centrifugation (4000 rpm; 5 min), the supernatant was collected and concentrated to approximately one-quarter of its original volume. The concentrated solution was deproteinized using the Sevag reagent method. Anhydrous ethanol was then added to the solution until the ethanol concentration reached 80% (*v*/*v*), and the mixture was refrigerated at 4 °C for 12 h. Afterward, the mixture was centrifuged at 5000 rpm for 10 min, and the sediment was washed with anhydrous ethanol and acetone. The resulting product was re-dissolved in deionized water and freeze-dried to obtain the polysaccharide, named W-RTFPs.

### 2.6. Structural Characterization

#### 2.6.1. Chemical Composition Analysis

The chemical composition analysis was carried out based on previous reports. The total sugar contents of the RTFPs and W-RTFPs were determined using the phenol–sulfuric acid method [10]. The protein contents of the RTFPs and W-RTFPs were measured using the Coomassie brilliant blue method [11]. The uronic acid contents of the RTFPs and W-RTFPs were determined by the m-hydroxybiphenyl colorimetry method [12].

#### 2.6.2. Molecular Weight Distribution Analysis

The molecular weight (Mw) profiles of the RTFPs and W-RTFPs were analyzed using high-performance liquid chromatography (HPLC; Agilent A1260 series, Agilent Technologies, Santa Clara, CA, USA) coupled with tandem gel permeation columns (G-5000 PWXL and G-3000 PWXL, both 7.8 × 300 mm). Samples (5 mg) were dissolved in 0.02 M phosphate buffer (pH 6.8), filtered through 0.22 μm membranes, and injected (20 μL) under the following conditions: isocratic elution with 0.02 M phosphate buffer at 0.6 mL/min, column temperature 35 °C. A differential refractive index detector quantified elution volumes, calibrated against pullulan standards (5.9–708 kDa) to establish a logarithmic Mw–elution volume relationship [9].

#### 2.6.3. Monosaccharide Composition Analysis

The monosaccharide composition profiles of RTFPs and W-RTFPs were analyzed using ion-exchange chromatography with pulsed amperometric detection (HPAEC-PAD; ICS-6000, Thermo Fisher Scientific, Sunnyvale, CA, USA). Each sample (5 mg) was dissolved in 4 mL of 2 M trifluoroacetic acid and hydrolyzed in a sealed ampoule at 105 °C for 6 h. After cooling, the residual trifluoroacetic acid was removed under reduced pressure at 45 °C. The resulting residue was dissolved in 4 mL of methanol and subjected to repeated reduced-pressure drying six times. The final residue was dissolved in deionized water and adjusted to a final volume of 50 mL. After filtration through a 0.22 μm filter, the filtrate was analyzed under the following conditions: a Carbopac PA20 chromatographic column (2 × 250 mm, 5 μm) coupled with an electrochemical detector, with a column temperature at 30 °C, a flow rate of 0.5 mL/min, and a sample injection volume of 20 μL. The mobile phase gradient elution was programmed as follows: 10% 20 mM NaOH for 0–16.05 min, followed by a mixture of 10% NaOH solution (20 mM) and 20% CH_3_COONa solution (500 mM) for 16.05–30.05 min. Monosaccharide standards were analyzed under identical conditions. Calibration curves were constructed by plotting the peak area (y) against the concentration (x) of monosaccharide standards to quantify the compositions.

#### 2.6.4. Fourier Transform Infrared Spectroscopy (FT-IR) Analysis

Functional groups were characterized using a Nicolet IS50 FT-IR spectrometer (Thermo Fisher Scientific, Waltham, MA, USA). Each sample (3 mg) was homogenously mixed with spectroscopic-grade KBr (200 mg), pelletized to a thickness of 1 mm, and scanned (64 scans, 500–4000 cm^−1^, 2 cm^−1^ resolution). Background correction was performed using a pure KBr reference.

#### 2.6.5. Iodine–Potassium Iodide (I_2_/KI) Binding Assay

Conformational analysis of polysaccharide samples was performed by mixing 2 mL of the sample solution (2.0 mg/mL) with 8 mL of the I_2_/KI reagent (0.02% I_2_ + 0.2% KI). After 10 min of equilibration, UV–Vis spectra of the sample (300–700 nm) were recorded using a Nano Ready F-1100 spectrophotometer (Meite Instrument Co., Ltd., Shanghai, China) with a resolution of 1 nm.

### 2.7. Antioxidant Capacity Assessment

#### 2.7.1. DPPH Radical Scavenging Activity Assay

The antioxidant potentials of RTFPs and W-RTFPs were evaluated using the DPPH radical scavenging assay [13]. Serial dilutions of the polysaccharide sample (12.5–400 μg/mL) were prepared in 0.02 M phosphate buffer (pH 7.4). The test solution was prepared by mixing 100 μL of the polysaccharide solution with 100 μL of a 0.2 mM DPPH methanolic solution (70% *v*/*v* methanol). After a 30 min incubation in the dark at 25 °C, the absorbance was measured at 517 nm using a microplate reader (Multiskan, Thermo Scientific, Waltham, MA, USA). Ascorbic acid (V_C_) was used as the positive control. The DPPH radical scavenging rate (%) was calculated as the following Equation (2):

(2) $$\mathrm{Scavenging}\;\mathrm{rate}\;(\%)\;=\left(1-\frac{A_{a\;}-{\;A}_{b}}{A_{c}}\right)\;\times \;100\%$$

where A_a_ is the absorbance of the sample and DPPH solution, A_b_ is the absorbance of 70% methanol and the sample, and A_c_ is the absorbance of the DPPH solution and 70% methanol.

#### 2.7.2. ABTS Radical Scavenging Assay

The ABTS radical scavenging capacities of RTFPs and W-RTFPs were assessed [14]. The ABTS^+^ stock solution was prepared by reacting 5 mL of 7 mM ABTS with 5 mL of 2.45 mM K_2_S_2_O_8_. Prior to analysis, the solution was diluted with PBS (pH 7.4) to achieve an absorbance of 0.70 ± 0.02 at 734 nm. The test sample (50–800 μg/mL, 200 μL) was mixed with 1 mL of the ABTS^+^ working solution, vortex-mixed, and incubated in the dark for 10 min at 25 °C. Absorbance was recorded at 734 nm using a microplate reader (Multiskan, Thermo Scientific, Waltham, MA, USA). Ascorbic acid (V_C_) served as the positive control. The radical scavenging rate (%) was calculated as the following Equation (3):

(3) $$\mathrm{Scavenging}\;\mathrm{rate}\;(\%)=\left(1-\frac{A_{1}}{A_{0}}\right)\;\times \;100\%$$

where A_0_ is the absorbance of the ABTS^+^ solution and PBS, and A_1_ is the absorbance of the ABTS^+^ solution and sample.

### 2.8. Immunomodulatory Activity Analysis

#### 2.8.1. Cell Culture

RAW264.7 murine macrophages (American Type Culture Collection, Rockville, MD, USA) were maintained in DMEM supplemented with 10% fetal bovine serum and 1% penicillin–streptomycin under 5% CO_2_ at 37 °C.

#### 2.8.2. Cytotoxicity Assay

Cell viability was quantified using the CCK-8 assay. Cells (1.8 × 10^4^ cells/well) were seeded in 96-well plates, allowed to adhere for 24 h, and then exposed to polysaccharide solution (100–400 μg/mL) in serum-free DMEM. After 24 h of treatment, 100 μL of a 10% CCK-8 reagent (composed of complete medium and CCK8 solution in a 10:1 ratio) was added to each well and incubated for 2 h. Absorbance was measured at 570 nm using a SpectraMax i3x microplate reader (Molecular Devices, San Jose, CA, USA). The cell viability rate was calculated using the following Equation (4):

(4) $$Cell\;\mathrm{viability}\;\mathrm{rate}\;(\%)=\frac{{\mathrm{OD}}_{a}\;-\;{\mathrm{OD}}_{b}}{{\mathrm{OD}}_{c}\;-\;{\mathrm{OD}}_{b}}\;\times \;100\%\;$$

where OD_a_ is the absorbance of the experimental group, OD_b_ is the absorbance of the blank control, and OD_c_ is the absorbance of the control group.

#### 2.8.3. Phagocytic Capacity Assay

Cells (2.0 × 10^4^ cells/well) were seeded in 96-well plates and cultured for 24 h. After washing with PBS three times, the cells were incubated with 200 µL of RTFPs and W-RTFPs at concentrations of 100, 200, and 400 μg/mL. The blank group received 200 µL of complete culture medium, while the control group received 200 µL of complete culture medium supplemented with 1 μg/mL LPS. After 24 h of incubation, the medium was replaced with 100 µL of 0.075% neutral red solution and incubated for 1 h. The cells were then washed with PBS three times, followed by the addition of 150 μL of cell lysate (ethanol/acetic acid = 1:1, *v*/*v*) into each well. After another hour, the absorbance of the supernatant was measured at 540 nm. The phagocytosis capacity was calculated using the following Equation (5):

(5) $$\mathrm{Phagocytosis}\;\mathrm{capacity}\;(\%)=\frac{{\mathrm{OD}}_{a}\;-\;{\mathrm{OD}}_{b}}{{\mathrm{OD}}_{c}\;-\;{\mathrm{OD}}_{b}}\;\times \;100\%\;$$

where OD_a_ is the absorbance of the sample group, OD_b_ is the absorbance of the blank group, and OD_c_ is the absorbance of the control group.

#### 2.8.4. Measurement of Cytokines

Cells (2.0 × 10^4^ cells/well) were seeded in 96-well plates and cultured for 24 h. The cells were then treated with 200 μL of RTFPs and W-RTFPs solutions at concentrations of 100, 200, and 400 μg/mL, and incubated for an additional 24 h. After incubation, the supernatants were collected, and the concentrations of NO and TNF-α were measured using Griess reagent and ELISA kits, according to the manufacturer’s instructions.

### 2.9. Data Analysis

Data were expressed as the means ± standard deviations (SDs). SPSS 13.0 software was used to conduct one-way analysis of variance (one-way ANOVA) and Duncan tests for difference significance analyses. When the significance level *p* < 0.05, the groups were considered different.

## 3. Results and Discussion

### 3.1. Effect of Extraction Conditions on the Yield of RTFPs

**Figure 1:**
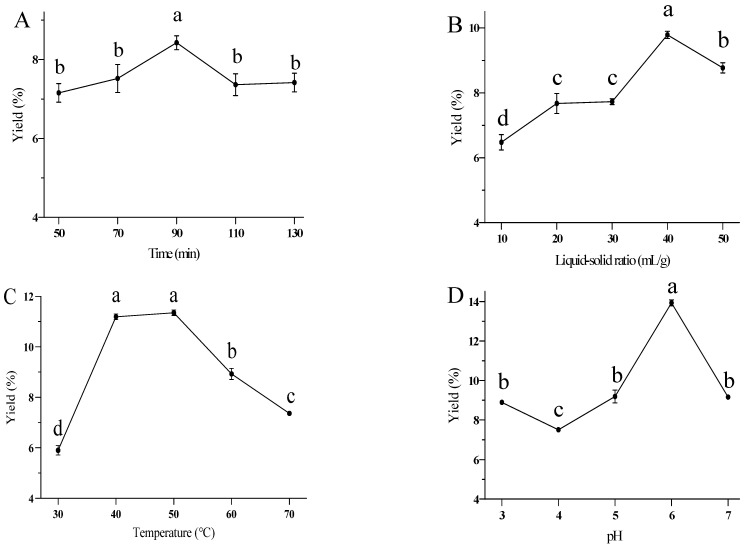
(**A**) Effects of extraction time, (**B**) liquid–solid ratio, (**C**) extraction temperature, and (**D**) extraction pH on the yield of RTFPs. The RTFPs were extracted from *R. roxburghii* fruit using an enzyme-assisted extraction method. Different letters indicate significant differences at a significance level of *p* < 0.05.

Figure 1A illustrates the effect of extraction time on the yield of RTFPs. The extraction yield of RTFPs at different extraction times (50, 70, 90, 110, 130 min) did not change significantly. The yield of RTFPs significantly increased from 50 min to 90 min, peaking at 90 min (*p* < 0.05). However, prolonged extraction time resulted in a decrease in yield, which stabilized around 130 min. This might be because a moderate increase in extraction time facilitated the effective breakdown of the substrate by cellulase, enhancing polysaccharide release. However, excessive extraction time could potentially degrade polysaccharides [6,15], thereby reducing the total yield. Therefore, 90 min was directly selected as the optimal extraction time without performing further optimization experiments.

Figure 1B shows the effect of the liquid-to-solid ratio on the yield of RTFPs. The RTFP yield significantly increased from 10 to 40 mL/g, but then decreased from 40 to 50 mL/g (*p* < 0.05). It is commonly believed that increasing the liquid-to-solid ratio enhances the interaction between the solvent and the sample, as well as the diffusion of polysaccharides, thereby boosting the extraction yield. However, at very high liquid-to-solid ratios, the lower concentration of substrate may reduce cellulase activity, leading to decreased breakdown of plant cell walls and a lower extraction yield [16]. Thus, the liquid-to-solid ratio was chosen to be within the range of 30 mL/g to 50 mL/g for the optimization experiment.

Figure 1C illustrates the effect of temperature on the yield of RTFPs. The yield initially significantly increased with rising temperature (*p* < 0.05), then decreased after reaching a peak at 50 °C. Notably, there was no significant increase in yield at 50 °C compared to 40 °C. A moderate increase in temperature enhances molecular diffusion and accelerates the transfer of intracellular substances. However, excessive temperature can damage the cellulase activity, reducing extraction yields [17]. Thus, the temperature was chosen to be within the range of 40 to 60 °C for the optimization experiment.

Figure 1D shows the effect of extraction pH on the yield of RTFPs. As extraction pH increased, the yield of RTFPs significantly increased between pH 4.0 and 6.0, peaking at pH 6.0, and then decreased beyond pH 6.0 (*p* < 0.05). Extreme pH levels adversely affect enzyme conformation and activity, thereby reducing the yield of RTFPs [18]. Thus, the extraction pH was chosen to be within the range of 5 to 7 for the optimization experiment.

### 3.2. Optimization of the Extraction Process

#### 3.2.1. Statistical Analysis and Model Fitting

The extraction liquid–solid ratio, pH, and temperature were used as independent variables. The yield of RTFPs was used as a response variable. The design matrix and corresponding results are presented in Table 2. Based on multiple regression analysis of the experimental data, the response variable and test variables were expressed by the following second-order polynomial Equation (6):

Yield (%) = 13.65 − 0.5462A − 0.6987B − 1.05C + 0.3525AB − 0.4400AC + 1.02BC − 1.87A^2^ − 2.22B^2^ − 1.69C^2^ (6)

The significance of the model was verified by analysis of variance (ANOVA) for the response surface quadratic model and the results are summarized in Table 3. The *p*-value of the model was 0.0003, indicating that the model was highly significant. Lack-of-fit is an important statistical metric used in RSM to assess the goodness-of-fit of the model. It is employed to test whether the model adequately fits the experimental data, specifically to determine if key variables have been omitted or if there are unaccounted interactions/nonlinear relationships. The lack-of-fit *p*-value was 0.2053 (*p* > 0.05), suggesting that the model fits well, with the residuals primarily arising from random errors rather than model deficiencies [19]. The *R*^2^ of 0.9647 and *R*^2^_Adj_ of 0.9193 of the model indicated a strong linear relationship between the dependent and independent variables, further confirming that the regression equation was suitable for analyzing the test results [15]. The established *R*^2^_Pre_ of 11.77, exceeding 4, confirmed that the signal-to-noise ratio was adequate [19]. The low coefficient of variation (C.V.% = 5.68%) indicated the quite high precision and reliability of the experimental values [20]. Based on the variance analysis, the primary terms (A, B, C) and quadratic terms (A^2^, B^2^, and C^2^) had significant effects on the yield of RTFPs (*p* < 0.05). According to the F-value of the primary terms, the factors influencing the yield of RTFPs were C > B > A. Thus, the model was significantly used to analyze the yield of RTFPs.

#### 3.2.2. Response Surface Analysis

**Figure 2:**
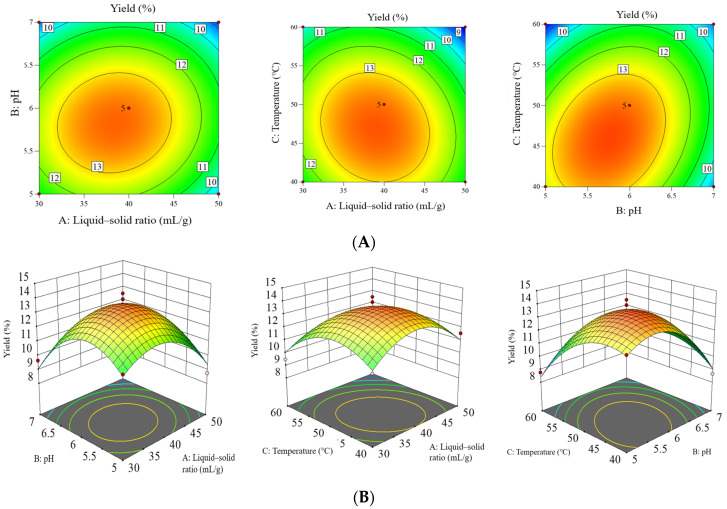
(**A**) Contour plots for effects of interaction between various factors on the yield of RTFPs and (**B**) response surface plots for effects of interaction between various factors on the yield of RTFPs. The RTFPs were extracted from *R. roxburghii* fruit using an enzyme-assisted extraction method.

The 3D response surfaces and 2D contour plots are effective tools for examining the interaction of different factors, as well as for analyzing the relationship between the responses and each tested variable [21]. As depicted in Figure 2, the yield of RTFPs initially increased and then decreased as the solid–liquid ratio, pH value, and extraction temperature were raised. This trend aligned with the results from the single-factor tests. The contour maps of AB, AC, and BC exhibited oval shapes, and all 3D response curves displayed slopes and maximum values, indicating significant interaction effects among the independent variables on the yield of RTFPs. The steepness of the 3D response surface and the ellipticity of the 2D contour plots followed the order BC > AC > AB. This suggests that the interaction between extraction pH and temperature had a more pronounced effect on the yield of RTFPs compared to other factor interactions, which is consistent with the results of the variance analysis presented in Table 3.

#### 3.2.3. Optimization and Validation of the Model

Response surface methodology was used to optimize and predict the experimental data. The optimal extraction conditions were as follows: liquid-to-solid ratio 38.7347 mL/g, extraction pH 5.7474, and extraction temperature 46.3152 °C, with a predicted RTFP yield of 13.9611%. According to the actual operation, the confirmatory extraction conditions were slightly adjusted to a liquid-solid ratio of 38.7 mL/g, an extraction pH of 5.75, and an extraction temperature of 46 °C. Under these adjusted conditions, the yield of RTFPs was 14.02%, which differed by only 0.43% from the predicted value of the model. This confirmed that the response surface regression equation effectively simulated the experimental process and provided accurate yield predictions for RTFPs. The established enzyme-assisted method was compared with the conventional hot water extraction method. The observed yield of RTFPs (14.02%) was significantly higher than that of W-RTFPs (3.98%). These results indicate that the enzyme-assisted extraction method substantially enhances the RTFP yield compared to the conventional hot water extraction method. Chen et al. extracted polysaccharides from *R. roxburghii* using ultrasound-assisted extraction, with a yield of 6.59 ± 1.34% [22]. In comparison, enzyme-assisted extraction of polysaccharides *from R. roxburghii* exhibits greater advantages.

### 3.3. Chemical Composition

The chemical compositions of RTFPs and W-RTFPs were comparatively analyzed using colorimetric methods. Quantitative assessments revealed distinct compositional profiles. As shown in Table 4, RTFPs exhibited a neutral sugar content of 36.38%, significantly lower than the 50.45% observed in W-RTFPs. In contrast, the uronic acid content displayed an inverse trend, with RTFPs containing 48.83%, substantially higher than the 39.19 ± 1.03% detected in W-RTFPs. The difference is likely due to the thermal degradation of uronic acid during hot water extraction. Additionally, small amounts of protein were detected in both RTFPs (7.29%) and W-RTFPs (6.58%). These results indicate that RTFPs have a higher uronic acid content compared to W-RTFPs. Overall, these findings suggest that the enzyme-assisted extraction method significantly influences the biochemical characteristics of RTFPs.

### 3.4. Structural Characterization of RTFPs and W-RTFPs

#### 3.4.1. Mw Distribution Profiles

**Figure 3:**
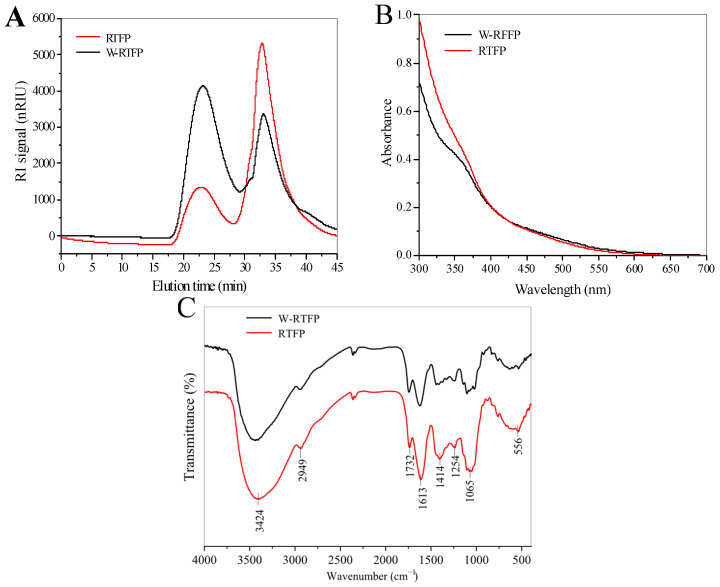
(**A**) Molecular weight distributions of RTFPs and W-RTFPs, (**B**) UV–vis spectra, and (**C**) FT-IR spectra of W-RTFPs and RTFPs. RTFPs and W-RTFPs were the *R. roxburghii* fruit polysaccharides obtained using an enzyme-assisted extraction and hot water extraction, respectively.

The Mw distribution profiles of RTFPs and W-RTFPs are shown in Figure 3A. Both RTFPs and W-RTFPs displayed two symmetric peaks, indicating that *R. roxburghii* polysaccharides consisted of two components with different Mws. According to the calibration curve equation (LogMw = −0.0008X^3^ + 0.0698X^2^ − 2.1692X + 27.963), the average Mw of the RTFPs was 1.87 × 10^5^ Da (24.6%) and 4.75 × 10^3^ Da (75.4%), while the Mw of the W-RTFPs was 1.65 × 10^5^ Da (55.7%) and 4.33 × 10^3^ Da (44.3%). These results suggest that the enzyme-assisted extraction method leads to a reduction in the Mw of RTFPs compared to the conventional hot water extraction method, a trend also observed in Korean ginseng polysaccharides [23]. Unlike the random chain scission induced by hot water, cellulase is able to specifically cleave the *β*-1–4-glycosidic bonds, resulting in a large amount of polysaccharides with lower and more homogenous Mws. Since the Mw of polysaccharides is closely related to their biological activities and functions, these differences imply that RTFPs and W-RTFPs may have distinct biological properties and potential application areas.

#### 3.4.2. I_2_-KI Analysis

The I_2_-KI test is commonly used to assess the presence of long, multi-branched structures in polysaccharides. When I_2_ reacts with polysaccharides that have fewer branches and shorter side chains, the UV absorption peak of the mixture shifts from 350 nm to 565 nm [24]. As shown in Figure 3B, neither W-RTFPs nor RTFPs exhibited an absorption peak at 565 nm in the KI-I_2_ test, indicating the presence of long side chains and branched structures in both samples.

#### 3.4.3. FT-IR Spectroscopy

FT-IR spectroscopy was used to analyze the main functional groups in the chemical structures of RTFPs and W-RTFPs; the results are presented in Figure 3C. Both RTFPs and W-RTFPs exhibited similar FT-IR spectral patterns. A weak absorption peak around 2949 cm^−1^ was attributed to the C-H stretching vibration [25], while the absorption peak near 1414 cm^−1^ corresponded to the deformation vibration of C-H bonds [26]. The absorption peak at approximately 1065 cm^−1^ was assigned to the stretching vibration of the pyranose ring [27]. However, variations in the intensity of certain characteristic bands highlight the effect of enzyme-assisted extraction on the structure of polysaccharides. RTFPs exhibited a broader absorption peak around 3424 cm^−1^, which can be attributed to O-H stretching vibrations. This suggests that RTFPs may contain more hydroxyl groups than W-RTFPs [28]. The absorption peak near 1613 cm^−1^, related to the C=O asymmetric stretching vibration of the carboxyl group, indicated the presence of uronic acid in the polysaccharide samples. This peak was stronger in RTFPs than in W-RTFPs, suggesting a higher content of uronic acid in RTFPs [26,29]. These results indicate that RTFPs exhibited typical absorption peaks for polysaccharides.

#### 3.4.4. Monosaccharide Composition

**Figure 4:**
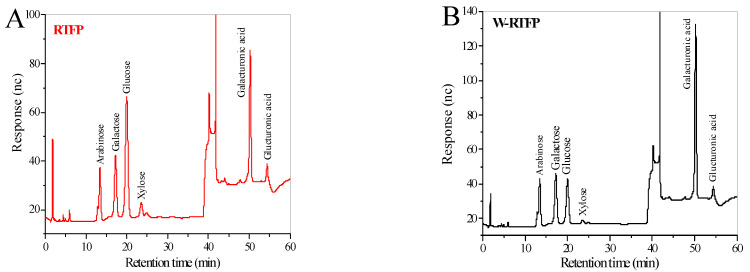
Monosaccharide composition chromatograms of (**A**) RTFPs and (**B**) W-RTFPs. RTFPs and W-RTFPs were the *R. roxburghii* fruit polysaccharides obtained using an enzyme-assisted extraction and hot water extraction, respectively.

The monosaccharide compositions of RTFPs and W-RTFPs were analyzed using ion-exchange chromatography, and the molar ratio percentages of each compositional monosaccharide were calculated based on the calibration curve equations for each monosaccharide standard. As shown in Figure 4, both RTFPs and W-RTFPs were found to contain arabinose, galactose, glucose, xylose, galacturonic acid, and glucuronic acid. RTFPs were composed of arabinose (20.66%), galactose (20.58%), glucose (38.93%), galacturonic acid (10.94%), xylose (6.52%), and glucuronic acid (2.27%) with the respective molar ratio of 9.10:9.06:17.15:4.82:2.93:1.00, while W-RTFPs were composed of arabinose (28.92%), galactose (25.41%), glucose (19.30%), galacturonic acid (22.65%), glucuronic acid (2.27%), and xylose (1.34%) with the respective molar ratio of 21.58:18.96:14.40:16.90:1.69:1.00. These results indicate that while the types of monosaccharides present were unaffected by the different extraction methods, there were notable differences in their relative concentrations. Such variations may change the functional and biological properties of RTFPs.

### 3.5. Antioxidant Activities of RTFPs and W-RTFPs in Vitro

**Figure 5:**
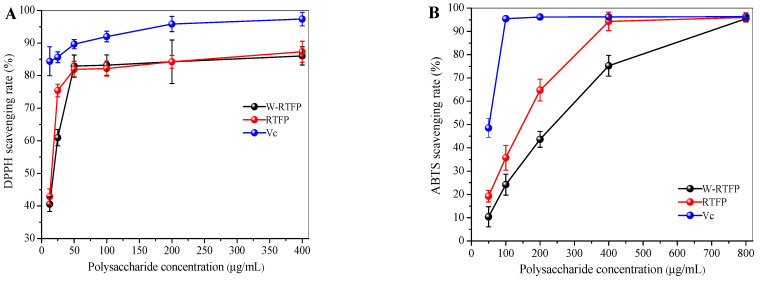
(**A**) DPPH and (**B**) ABTS free radical scavenging capacities of RTFPs and W-RTFPs. RTFPs and W-RTFPs were the *R. roxburghii* fruit polysaccharides obtained using an enzyme-assisted extraction and hot water extraction, respectively. Vc was used as the positive control.

The antioxidant capacities of RTFPs and W-RTFPs were evaluated using DPPH and ABTS radical scavenging assays [30,31,32,33,34]. In the DPPH assay, antioxidants donate electrons or hydrogen atoms to neutralize the stable DPPH radical, resulting in a visible reduction in absorbance proportional to their scavenging activity. As shown in Figure 5A, both RTFPs and W-RTFPs exhibited concentration-dependent DPPH radical scavenging effects within the 0–50 μg/mL range, achieving scavenging rates exceeding 80% at 50 μg/mL. Notably, their performances matched that of vitamin C (V_C_) at equivalent concentrations. The calculated IC50 values further highlighted RTFPs’ superior efficacy (11 μg/mL) compared to that of W-RTFPs (15 μg/mL).

The ABTS assay corroborated these findings (Figure 5B). Over a broader concentration range (12.5–400 μg/mL), RTFPs demonstrated a dose-responsive increase in scavenging activity, reaching 94.31% at 400 μg/mL—comparable to V_C_. Consistent with the DPPH results, RTFPs exhibited a significantly lower IC50 value of 150 μg/mL than W-RTFPs, confirming that enzymatic-assisted extraction enhances the antioxidant potential of *R. roxburghii* fruit polysaccharides. This aligns with previous reports on enzymatic extraction improving bioactivity in other polysaccharides, such as those from *Polygonatum odoratum* [9].

The enhanced antioxidant performance of RTFPs may be due to their structural characteristics: (1) their lower molecular weight (Mw), which increases the accessibility of reductive hydroxyl termini to neutralize free radicals, (2) their greater number of hydroxyl groups, which provide hydrogen atoms or electrons to bind with free radicals at the C(O)-2 and C(O)-3 sites, and (3) their higher uronic acid content. The electrophilic keto and aldehyde groups in uronic acids facilitate hydrogen atom dissociation from O–H bonds, thereby amplifying radical scavenging efficiency [13,26,35]. In contrast, W-RTFPs’ greater Mw likely restricts conformational flexibility, limiting exposure of their antioxidant functional groups [36]. These structure–activity relationships underscore the critical role of extraction methods in modulating polysaccharide bioactivity. The collective data demonstrate that enzymatic extraction not only alters the chemical composition of RTFPs but also optimizes their molecular architecture for enhanced antioxidant functionality, positioning them as promising bioactive agents for further investigation.

### 3.6. Cytotoxicities of RTFPs and W-RTFPs

**Figure 6:**
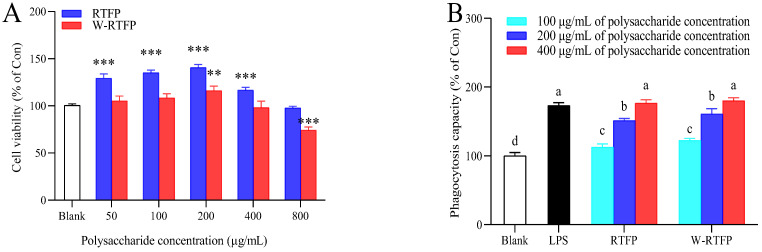
Effects of RTFPs and W-RTFPs on the (**A**) cell viability and (**B**) phagocytic capacity of RAW264.7 cells. RTFPs and W-RTFPs were the *R. roxburghii* fruit polysaccharides obtained using an enzyme-assisted extraction and hot water extraction, respectively. Lipopolysaccharide (LPS) was used as the positive control. ** indicates significant difference between control group and experimental groups (*p* < 0.01); *** indicates extremely significant difference between control group and experimental groups (*p* < 0.001). Different letters indicate significant differences between different components (*p* < 0.05).

The cytotoxic potentials of RTFPs and W-RTFPs were evaluated in murine macrophage RAW 264.7 cells using a CCK-8 assay following 24 h exposure to polysaccharide concentrations ranging from 50 to 800 μg/mL. As illustrated in Figure 6A, neither polysaccharide fraction induced significant cytotoxicity at the tested concentrations. Strikingly, RTFPs exhibited dose-dependent proliferative effects, enhancing cell viability by up to 128% within the 50–400 μg/mL range (*p* < 0.001), indicative of superior biocompatibility relative to W-RTFP-treated cells. A concentration threshold was observed at 800 μg/mL, where mild cytotoxicity emerged, particularly in W-RTFP-treated cells (*p* < 0.05). Based on these findings, non-cytotoxic concentrations of 100, 200, and 400 μg/mL were selected for subsequent immunoassays to ensure biological relevance while avoiding interference from cell viability artifacts.

### 3.7. Effects of RTFPs and W-RTFPs on Macrophage Phagocytic Activity

Macrophages, specialized immune cells capable of engulfing pathogens, cellular debris, and apoptotic cells, play a critical role in innate immunity [37]. Enhanced phagocytic activity serves as a hallmark of macrophage activation, reflecting their functional responsiveness [38]. As illustrated in Figure 6B, both RTFPs and W-RTFPs dose-dependently augmented phagocytosis in macrophages across a concentration range of 100–400 μg/mL. Compared to untreated controls, RTFPs increased phagocytic activity by 12.61–76.63%, while W-RTFPs elicited a 22.27–80.17% enhancement. These results demonstrate that both polysaccharide fractions significantly stimulate macrophage phagocytosis, with W-RTFPs showing marginally greater efficacy at higher concentrations.

### 3.8. Effects of RTFPs and W-RTFPs on the Production of Cytokines

Activated macrophages secrete critical immunomodulatory mediators, including nitric oxide (NO) and tumor necrosis factor-alpha (TNF-α), which play pivotal roles in antimicrobial defense and tumor suppression [38,39,40,41,42,43]. NO acts as a key cytotoxic effector molecule, while TNF-α induces selective apoptosis in malignant cells while sparing healthy tissues [44,45]. These cytokines are widely used biomarkers for evaluating macrophage activation by polysaccharides.

**Figure 7:**
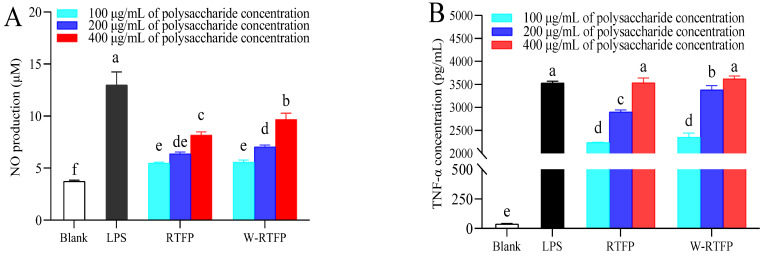
Effects of RTFPs and W-RTFPs on (**A**) NO and (**B**) TNF-α production in RAW264.7 cells. RTFPs and W-RTFPs were the *R. roxburghii* fruit polysaccharides obtained using an enzyme-assisted extraction and hot water extraction, respectively. Lipopolysaccharide (LPS) was used as the positive control. Different letters indicate significant differences between different components (*p* < 0.05).

As shown in Figure 7, untreated RAW264.7 cells exhibited minimal NO (A) and TNF-α (B) secretion. Both RTFPs and W-RTFPs stimulated dose-dependent increases in cytokine production (*p* < 0.001), aligning with established immunostimulatory mechanisms of polysaccharides [46]. However, distinct activity profiles emerged: RTFPs enhanced NO production by 46.87–119.32% and TNF-α by 5891.76–9386.09% across tested concentrations (100–400 μg/mL), whereas W-RTFPs induced broader increases of 49.19–159.97% (NO) and 6198.72–9614.26% (TNF-α). Notably, RTFPs showed reduced efficacy at higher doses, with significantly lower NO secretion than W-RTFPs at 400 μg/mL and diminished TNF-α stimulation at 200 μg/mL (*p* < 0.05).

The differential responses may correlate with structural recognition mechanisms. Polysaccharides have the potential to interact with macrophage pattern recognition receptors (PRRs)—such as TLR2/4, CR3, and mannose receptors—through specific structural motifs, including galactose and arabinose residues [37,38,47]. The presence of galactose, glucose, and arabinose in these polysaccharides possibly enhances recognition by cell membrane receptors, thereby promoting macrophage-mediated immune responses [48]. Notably, the higher arabinose/galactose content in W-RTFPs (Figure 4) is likely to improve PRR binding efficiency, which may explain its slightly enhanced cytokine induction. These findings are consistent with studies on *Hylocereus undatus* polysaccharides, where arabinose/galactose-rich fractions exhibited amplified immunomodulatory activity [37]. Future research will focus on receptor block experiments to further verify the immune molecular mechanisms underlying the activity of these polysaccharides. Moreover, polysaccharides with larger Mws (>100 kDa) generally exhibit higher immunomodulatory activities, offering a plausible explanation for the slightly stronger immune response stimulation observed with W-RTFPs [38,49]. These findings underscore the significant impact of polysaccharide composition and molecular weight on their immunomodulatory effects. Overall, both RTFPs and W-RTFPs exhibited potent immunomodulatory activities, which can be attributed to their moderate molecular weight distribution, unique monosaccharide profile, and highly branched conformation.

## 4. Conclusions

This study demonstrates that enzyme-assisted extraction is a highly efficient and scalable method for isolating bioactive polysaccharides from *Rosa roxburghii* Tratt fruit (RTFPs), offering a substantially higher yield compared to conventional hot water extraction. RTFPs exhibited potent antioxidant activity, comparable to standard antioxidants, along with dose-dependent immunomodulatory effects, including enhanced phagocytic capacity and elevated secretion of key immune mediators such as NO and TNF-α, reaching levels similar to those induced by positive controls. These findings provide critical insights into the structure–activity relationships of RTFPs, correlating their monosaccharide composition, molecular weight distribution, and structural features with their antioxidant and immunostimulatory properties. Beyond establishing an optimized extraction protocol, this study advances the understanding of RTFPs’ bioactivity, supporting their potential applications in functional food and pharmaceutical development. Future research should explore the underlying mechanisms of action and validate these effects through in vivo studies.

## Figures and Tables

**Table 1:** Box–Behnken design (BBD) variables and coded factors.

Factors	Symbol	Coding and Level		
		−1	0	1
Liquid–solid ratio (mL/g)	A	30	40	50
Extraction pH	B	5	6	7
Extraction temperature (°C)	C	40	50	60

**Table 2:** Box–Behnken design (BBD) and experimental results.

Run	A	B	C	Yield (%)
1	40	5	60	8.77
2	40	7	60	8.94
3	50	6	60	8.25
4	30	6	60	9.49
5	40	6	50	14.33
6	40	6	50	13.09
7	30	5	50	11.28
8	40	6	50	13.45
9	50	7	50	8.53
10	40	6	50	13.94
11	40	6	50	13.42
12	30	7	50	9.65
13	50	5	50	8.75
14	50	6	40	11.55
15	30	6	40	11.03
16	40	5	40	12.57
17	40	7	40	8.66

**Table 3:** Analysis of variance of quadratic regression equation.

Source	Sum of Squares	Degree of Freedom	Mean Square	*F* Value	*p* Value	Significance
Model	73.54	9	8.17	21.26	0.0003	**
A	2.39	1	2.39	6.21	0.0415	*
B	3.91	1	3.91	10.16	0.0153	*
C	8.74	1	8.74	22.73	0.0020	**
AB	0.4970	1	0.4970	1.29	0.2929	
AC	0.7744	1	0.7744	2.01	0.1987	
BC	4.16	1	4.16	10.83	0.0133	*
A^2^	14.79	1	14.79	38.48	0.0004	**
B^2^	20.74	1	20.74	53.96	0.0002	**
C^2^	12.05	1	12.05	31.35	0.0008	**
Residual	2.69	7	0.3843			
Lack-of-fit	1.74	3	0.5791	2.43	0.2053	
Pure Error	0.9529	4	0.2382			

* *p* < 0.05; ** *p* < 0.01.

**Table 4:** Chemical compositions of RTFPs and W-RTFPs *.

Fraction	RTFPs	W-RTFPs
Sugar content (%)	36.38 ± 0.45	50.45 ± 0.84%
Uronic acid content (%)	48.83 ± 0.53	39.19 ± 1.03
Protein content (%)	7.29 ± 0.38	6.58 ± 0.15

* RTFPs and W-RTFPs were the *R. roxburghii* fruit polysaccharides obtained using an enzyme-assisted extraction and hot water extraction, respectively.

## Data Availability

The original contributions presented in the study are included in the article; further inquiries can be directed to the corresponding author.
